# Menthol and flavor capsule cigarettes in the Philippines: A comparison of pack design

**DOI:** 10.18332/tid/112718

**Published:** 2019-11-01

**Authors:** Jennifer L. Brown, Katherine Clegg Smith, Meng Zhu, Meghan B. Moran, Connie Hoe, Joanna E. Cohen

**Affiliations:** 1 Department of Health, Behavior and Society, Johns Hopkins Bloomberg School of Public Health, Baltimore, United States; 2 Johns Hopkins Carey Business School, Baltimore, United States; 3 Department of International Health, Johns Hopkins Bloomberg School of Public Health, Baltimore, United States

**Keywords:** content analysis, tobacco marketing, tobacco product packaging

## Abstract

**INTRODUCTION:**

Tobacco use is a major public health problem in the Philippines. Menthol flavored and flavor capsule cigarettes are independently associated with increased smoking initiation and appeal to youth and young adults. Packaging is an important tobacco marketing tool. We describe cigarette packs sold in the Philippines market and describe products’ flavor and capsule inclusion.

**METHODS:**

Tobacco packs were systematically collected in the Philippines in 2016 and categorized as non-flavored non-capsule, menthol non-capsule, menthol capsule, and non-menthol capsule. Structural elements (e.g. pack type, shape) and graphic components (e.g. imagery, descriptors, color) of the packs were compared.

**RESULTS:**

Menthol capsule packs were significantly more likely to be hard packs than menthol non-capsule. Menthol packs were more likely to be colored green than non-flavored packs. Non-menthol capsule packs were more likely to display the term ‘fresh’ than non-capsule packs. Capsule packs were more likely to display technological appeals than non-capsule packs.

**CONCLUSIONS:**

Menthol and flavor capsule cigarettes are packaged differently (most notably, in terms of color and technological appeals) than non-menthol and non-capsule packs. Packaging and labeling policy should take this into consideration.

## INTRODUCTION

Tobacco use is the world’s leading cause of preventable death and the burden of the tobacco epidemic is increasingly falling on low- and middle-income countries^1^. Eighty per cent of tobacco-related deaths occur in low- and middle-income countries^2^. The Western Pacific region has historically been targeted by transnational tobacco companies^3,4^. In the Philippines, 22.7% of the adult population smokes (21.7% in urban and 23.2% in rural areas)^5^.

In all, 21.5% of the population in the Philippines smoke manufactured cigarettes, significantly higher than the use of hand-rolled cigarettes (2.5%) or kreteks (0.4%)^5^. The prevalence of smoking manufactured cigarettes in urban and rural areas does not vary significantly (21.3% and 21.7%, respectively)^5^. The estimated market share for menthol cigarettes in the Philippines varies by source, but was estimated to be 50% in 2010 by Philip Morris and 22% in 2017 by Euromonitor^6,7^. The Philippines is one of the top five menthol markets in the world and even though world market share is going down, the menthol market in the Philippines is increasing^7^. In addition, flavor capsule cigarettes — cigarettes that contain a liquid-filled capsule in the filter that can be crushed by the user to release a flavor — have penetrated the cigarette market in the Philippines in recent years^7,8^. Monitoring of cigarettes on the market in the Philippines indicates that three flavor capsule variants (FCVs) that are owned by two brand companies (Philip Morris Fortune Tobacco Corp and Japan Tobacco International) were available on the market in 2013, but by 2016, 16 FCVs that are owned by four companies (Philip Morris Fortune Tobacco Corp, Japan Tobacco International, British American Tobacco, and KT&G) were on the market^9^. While market share for flavor capsule cigarettes is still relatively low in the Philippines (4.1%)^7^, further market growth is expected^7,10^. Most flavor capsule cigarettes contain menthol, either as a menthol flavored cigarette with an added menthol capsule or as a non-flavored cigarette that contains a menthol flavored capsule that becomes menthol flavored when the capsule is crushed^7^. Less common, some flavor capsule cigarettes are fruit flavored or contain a non-characterizing flavor such as ‘purple’ or ‘ruby’ in addition to menthol flavoring^9^.

Menthol flavored tobacco products pose a unique threat to public health. Menthol flavoring can mask the harshness of smoking^7^ and menthol cigarettes are smoked disproportionately by vulnerable populations^11^, and are associated with increased smoking initiation^12^, increased likelihood of addiction^13^, and decreased likelihood of staying abstinent^14^. Research in high-income countries has found that some people, albeit a small percentage, still believe that menthol cigarettes are less harmful than non-menthol cigarettes^15^. One study, conducted in two upper middle-income countries, found that in Malaysia 16% of participants agreed that menthol cigarettes are less harmful than non-menthol cigarettes, while in Thailand 35% of participants agreed with this statement^16^. While limited research has been conducted on perceptions of flavor capsule cigarettes, early research has found that among youth flavor capsule cigarettes are perceived as less harmful and are associated with greater attractiveness and interest to try^17-19^. Young adults also have positive perceptions of flavor capsule cigarettes^20,21^. Adult smokers in the UK have reported using them because of the taste, smoothness, choice of flavors, and enjoyment associated with bursting the capsule^22^.

### Importance of packaging as a marketing tool

Packaging is a key marketing strategy. Some consider packaging to be the most important way that a marketer communicates with a potential consumer because it is present at the time the purchase is being made, and consumers may therefore interact with the packaging during purchase and use, and may look to the package for information on the product^23^. For cigarettes, packaging has also become more important over time as advertising, via media such as television and radio, becomes restricted^22,24^. In the Philippines, the Tobacco Regulation Act of 2003 bans tobacco advertising on domestic TV and radio, in domestic newspapers and magazines, as well as outdoors. The law, however, does allow tobacco advertising and promotion at the point-of-sale.

The cigarette package has been described by marketers as a ‘badge product’, meaning that cigarette companies use product design characteristics to get users to identify with the brand image, thus increasing brand loyalty^22^. Unlike some products where the packaging is discarded after opening, cigarettes are usually kept in their package until they are all smoked. The pack may be on display (such as out on a table) during the act of smoking, as well as beyond^25^. It is estimated that pack-a-day smokers may view the cigarette packaging up to 7000 times a year^26^. Consumers, including non-smokers, are also exposed to cigarette displays at point-of-sale, where cigarette packs can communicate information about a product to a wide audience.

Packaging is also used to differentiate between different brands and different cigarette products and plays a key role in influencing consumer decisions. Elements of the packaging, such as pack shape, opening, material, color, imagery, and descriptors, work in concert to communicate product characteristics to consumers^22^. Packaging effects are so strong that research shows consumer perceptions of cigarettes are altered based on packaging, independent of the product the packaging contains^22^.

### Marketing of menthol and flavor capsule cigarettes

The tobacco industry has historically targeted youth and minorities with menthol cigarette advertisements^27^. There is no research available on the marketing of menthol cigarettes in the Philippines specifically, but research on the US menthol market exists that may provide important insights. In the United States, marketing of menthol cigarettes using health reassurance messages was common until the mid-1900s. Following the 1950s, the tobacco industry shifted their message from focusing on health messages to focus on the ‘refreshing’ taste of menthol cigarettes and creating associations between menthol cigarettes and group identity, youthfulness, and fun^28,29^. While extensive research has been done on tobacco packaging design in general, there is no research specifically examining the differences between packaging of menthol and non-menthol cigarettes. Tobacco industry documents describe findings from the industry’s consumer perception studies that conclude that smokers prefer and associate green colored packaging with menthol cigarettes^30^.

No study has been published, to date, that focuses on the messages used to market flavor capsule cigarettes. Market research reports and observations do, however, provide some insight. Early market research by tobacco companies on flavor capsule cigarettes found that consumers like the control they have over the flavor of the cigarette and being able to decide when they can crush the capsule, as well as the sensations of feeling and hearing the capsule pop^31^. Therefore, advertising themes for flavor capsule cigarettes have included an emphasis on freshness and the option users have to customize their cigarette by having the choice to decide when to change the taste of the cigarette^8^. Slogans like ‘Click, Switch, Refresh’ and ‘Squeeze, Click, Change!’ have been used to promote flavor capsule cigarettes^8^. Many flavor capsule cigarette brands suggest menthol or mint flavoring through descriptors such as: ‘fresh’, ‘ice’, ‘frost’, ‘crisp’, ‘cold’ and ‘blast’^7^. Technology is generally used as a selling point in innovation and used to market flavor capsule cigarettes as well^7^. Flavor capsule advertising has also been described as ‘colorful, dynamic, and innovative’^8^.

### Objectives

Given how integral packaging is to marketing, it is important to understand how menthol and non-menthol cigarettes are presented via packaging. It is also important to differentiate between flavor capsule and non-flavor capsule packs, as many flavor capsule packs contain menthol and are rising in popularity^7,10^. Elucidating the differences in packaging design between different categories of packs will help us understand the differences in how packs are marketed by the tobacco industry. This, subsequently, will allow for exploration of the aspects of the packaging design that appeal to certain groups of consumers and influence consumer perceptions of these particular products. This will contribute to addressing consumer misperceptions and counter tobacco industry marketing tactics that attract new smokers.

The aim of our research was to compare and describe the similarities and differences in packaging components being used between capsule and non-capsule cigarette packs of varied flavors that are on the market in the Philippines. Specifically, we assessed: 1) the structural components (pack type, opening style, shape) that are used to package cigarettes; 2) the graphic components (color, imagery, descriptors) that are used on cigarette packaging; and 3) whether there are differences and/or similarities between packaging that varies by flavor and capsule presence, with respect to structural and graphic components.

## METHODS

### Design

We conducted a quantitative content analysis of cigarette packs purchased in the Philippines via the Tobacco Pack Surveillance System (TPackSS), Wave II data collection (November 2016). TPackSS is a surveillance study that aims to construct a sample of tobacco packaging in several low- and middle-income countries, including the Philippines, that is representative of the cigarette packaging available on the market at time of data collection^32^. Constructing the sample was done with the goal of maximizing diversity in terms of the cigarette packages collected. The TPackSS data collection protocol is publicly available at: http://globaltobaccocontrol.org/tpackss/ resources.

In the Philippines, data were collected from the most populous metropolitan area in the country, Metro Manila, and two of the remaining ten most populated cities in the country, Cebu and Davao. These cities were chosen based on population size, as well as geographical and cultural diversity. Within each city, twelve barangays (the smallest political units into which cities and municipalities are divided) were selected for sampling. Local partners in the Philippines constructed a sampling frame of barangays and classified them as low, middle, or high socioeconomic status, based on income and property tax information. In each city, four barangays each from the low, middle, and high socioeconomic strata, were selected purposively based on diverse geographical and residential composition.

Within each barangay, tobacco vendors were sampled. The types of vendors sampled were selected based on information from the Philippines Global Adult Tobacco Survey and Euromonitor country level data. Four types of tobacco vendors were purposively selected based on consumer purchasing and product distribution ranking among vendor types in the country. In the Philippines, the vendors selected were sari sari shops (small, locally owned neighborhood stores that sell a limited selection of groceries, home goods, snacks, and cigarettes), mall kiosks, convenience stores, and supermarkets. In each barangay, a hub (a transit center, major shopping center, or source of commerce) was selected where data collectors would start. Data collectors then used vendor selection information and a walking protocol to navigate to the first vendor. At the first vendor in the first barangay visited in the first city, data collectors purchased one of every unique cigarette pack available for sale. Unique cigarette packs were defined as any pack with at least one difference in an exterior feature of the pack, excluding health warning label and including but not limited to: stick count, size, brand name presentation, color, cellophane, packaging material (i.e. hard, soft, tin), and inclusion of a promotional item. In the subsequent barangays visited, one of every unique cigarette pack that was not already purchased at a previous vendor was purchased. If the selected vendor in a specific barangay did not have any new unique packs, data collectors visited up to three additional vendors in the barangay to find unique tobacco packs before proceeding to the next barangay. Data collectors kept track of the unique tobacco packs that had already been collected by taking pictures of the packs purchased and organizing them into brand folders on an iPad for easy cross-referencing.

### Coding

A codebook was developed based on the literature on cigarette marketing, branding, and audience segmentation and existing coding systems for tobacco packaging. Structural elements coded included features of packaging such as type (e.g. hard, soft, sachet), shape, size, and opening style. Graphic components coded included color, imagery, and descriptors. Imagery and descriptors were organized by the qualities or messages they connote, such as luxury, less harm, or femininity. The codebook used is publicly available at https://www.globaltobaccocontrol.org/tpackss/sites/default/files/Tobacco%20Packaging%20Features%20and%20Marketing%20Appeals%20Codebook%202017.pdf.

The definitions for all cigarette classifications, structural elements, and graphic components are found in Table 1. All sides of the cigarette package were considered during coding (as well as the larger package if the pack was contained within any additional packaging), and any cellophane wrapping, the inside of the packaging, packaging inserts, and the cigarette sticks.

**Table 1:** Definitions of key variables

*Variable*	*Definition*
Non-flavored	No indication that pack is flavored and no distinguishable flavor/taste/aroma other than tobacco is displayed on cigarette pack or stick.
Menthol flavor	‘Menthol’ or ‘mint’ appears as a descriptor on the cigarette pack or cigarette stick; includes flavors such as ‘purple menthol’.
Non-menthol flavor	A characterizing flavor descriptor, other than ‘menthol’ or ‘mint’, displayed as a descriptor on the cigarette pack or stick. Included, but not limited to caramel/vanilla/chocolate, cinnamon/canella or other spice, clove/kretek, fruit or citrus, coffee, alcoholic beverage, energy drink or an indication that cigarette is flavored, but no distinguishable flavor/taste/aroma (other than tobacco) is displayed on cigarette pack or stick.
Flavor capsule pack	Pack that indicates in any way that the user is able to change the stick flavor (e.g. convertibles, click and roll, activate freshness).
Traditional pack	Rectangular pack with a width to height ratio of 2:3.
Slim pack	Pack with a side width of 1.3 cm or less.
Hard pack	Pack with defined shape often constructed out of paper cardboard, which will hold its shape when sticks are removed, regardless of original shape.
Soft pack	Pack with malleable shape made of paper or cardboard with exposed foil or paper.
Principal color^a^	The dominant color on which other items are printed and/or a prominent color at first sight of the pack; up to two principal colors were identified per pack.
Feminine appeal	Includes descriptors such as flower terminology (roses, daisies etc.), fashion terms, synonyms for ‘slim’ (slender, skinny etc.), terms for women such as ‘lady’ or ‘girl’, ‘pink’ and/or images of items such as flowers/butterflies, fashion-related items, pink color, and a non-sexualized female form.
Less harm descriptors	Includes the descriptors, ‘light(s)’, ‘mild’, ‘low’, ‘safe(r)’, ‘smooth’, ‘soft’, ‘mellow’ and/or any qualitative description of the levels of nicotine, tar, or carbon monoxide, numbers potentially indicating strength, and any mention or long life or good health.
Technological appeal	Includes descriptors, ‘technology’, ‘less odor’, ‘odor reducing’, ‘less smoke smell’, ‘RELOC’, ‘resealable’, ‘adhesive’, ‘Pro Fresh’ and descriptors referring to a secondary technology (nano, high-definition, HD, system), terms referring to turning something off or on (switch, activate, click, press to refresh), terms indicating innovation (new, new generation, innovative, modern, advanced, progressive) and/or imagery such as power buttons, play buttons, skip track buttons, a ball illustration representing change in flavor (excluding buttons), and a stick filter.

a a The coders were given the following instructions: ‘First, select the background color of the primary package, i.e. the dominant color upon which other items are printed. Next, select a second color. Excluding the background color and the color of the brand name text, what is the one other main prominent color (if any)? If there is no other main color, select “no other main color”. The second color should be the other prominent and obvious color at first sight of the pack’. For the colors reported, per cent agreement between two coders ranged from 90.6–100%.

Packs were double coded by two trained research assistants. Intercoder reliability was assessed using per cent agreement and the prevalence-adjusted and bias-adjusted kappa statistic (PABAK). When results for all variables were averaged, we found a total observed agreement of 98.7% (95% CI: 96.82–99.91) and a PABAK of 0.973 (95% CI: 0.936–0.998). These statistics indicate near perfect agreement. Discrepancies were reviewed and resolved by a third trained coder.

### Sample

The sample of packs included in this study was limited to legal cigarettes displaying a Philippines health warning label in rotation at the time of data collection. A total of 158 packs were collected in the Philippines; 40 were excluded for being duplicate packs, 11 were excluded for being promotional items or roll-your-own cigarettes, two packs were excluded for being illicit, and 30 packs were excluded because they displayed a previously rotated health warning; 75 cigarette packs fit the inclusion criteria.

The 75 packs were manufactured by the brand owners: Associated Anglo American Tobacco Corporation (n=2), British American Tobacco (n=5), Japan Tobacco International (n=14), Kenstand Philippines Inc. (n=2), Korean Tobacco and Ginseng Corporation (n=6), Mighty Corporation (n=14), and Philip Morris International Inc. (n=32).

### Data analysis

Data analyses were conducted using Stata 14. Descriptive statistics were estimated and packs were categorized into four groups based on cigarette flavor and flavor capsule presence: 1) non-flavored non-capsule, 2) menthol non-capsule, 3) menthol capsule, and 4) non-menthol capsule (defined in Table 1). The structural elements and graphic components of the cigarette packaging were assessed by packaging category using Fisher’s exact test. If the differences in the proportions were significant according to Fisher’s exact test, pairwise comparisons were used to compare individual groups and the Bonferroni correction was applied to adjust for multiple comparisons.

## RESULTS

**Figure 1:**
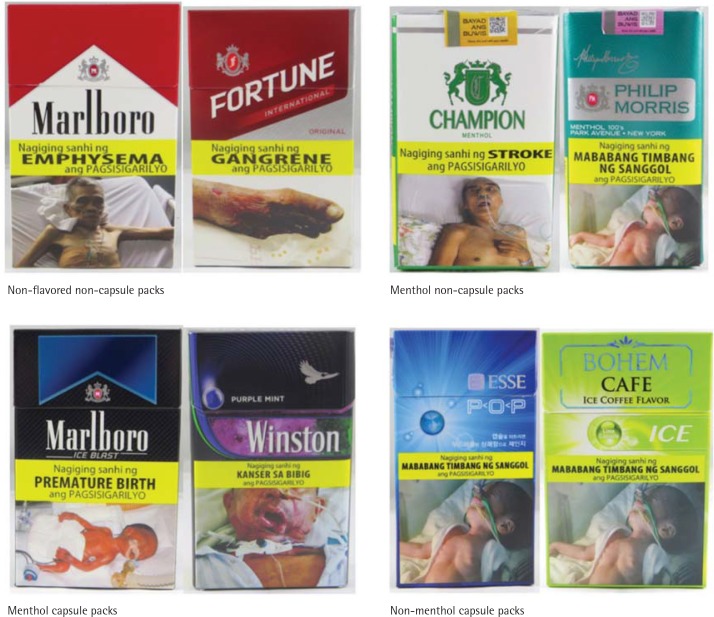
Examples of groups of packs by flavor and capsule presence

Of the 75 cigarette packs, 36 (48.7%) were non-flavored with no capsule, 23 (30.3%) were menthol flavored with no capsule, 10 (13.1%) were menthol with one or more capsules, and six (7.9%) were a non-menthol flavor with one or more capsules. Of the six non-menthol packs, four indicated that they included flavoring but did not display a characterizing flavor, one was ‘ice coffee flavor’ with a ‘lime’ capsule, and one was ‘orange coffee flavor’ with an ‘orange’ capsule. Figure 1 gives examples of packs grouped by flavor and flavor capsule inclusion.

### Structural elements

Overall, the groups of packs did not vary much with regard to structural elements (Table 2). All packs across all categories were traditionally shaped (rectangular pack with width to height ratio of 2:3). Five packs (6.6%) were slim packs, however there was no significant difference in the proportion of slim packs across pack groups. Overall, 54 packs (72.0%) were hard and 21 packs (28.0%) were soft. A significantly greater proportion of menthol capsule packs (n=10; 100%) were hard than were menthol non-capsule packs (n=11; 47.8%) (p=0.005).

**Table 2:** Structural elements of cigarette packaging by packaging category, as distinguished by flavor and flavor capsule presence

	*Group 1 Non-flavored non-capsule (n=36) n (%)*	*Group 2 Menthol non-capsule (n=23) n (%)*	*Group 3 Menthol capsule (n=10) n (%)*	*Group 4 Non-menthol capsule (n=6) n (%)*	*Groups 1 vs 2 p*	*Groups 1 vs 3 p*	*Groups 1 vs 4 p*	*Groups 2 vs 3 p*	*Groups 2 vs 4 p*	*Groups 3 vs 4 p*
**Pack type^a^**					0.051	0.172	0.312	0.005*	0.028	-
Hard	27 (75)	11 (47.8)	10 (100)	6 (100)	n/a	n/a	n/a	n/a	n/a	n/a
Soft	9 (25.0)	12 (52.2)	0 (0)	0 (0)	n/a	n/a	n/a	n/a	n/a	n/a
Traditional shape	36 (100)	23 (100)	10 (100)	6 (100)	-	-	-	-	-	-
Slim pack	2 (5.6)	1 (4.3)	0 (0)	2 (33.3)	1.000	1.000	0.091	1.000	0.100	0.125

* p<0.008

a critical value of 0.008 was used to assess whether any pairwise comparison was considered statistically significant in order to account for multiple comparisons and control the family-wise error rate. a For pack type, a test of the two-way table was conducted. A dash (-) indicates no difference between groups.

### Graphic components

**Figure 2:**
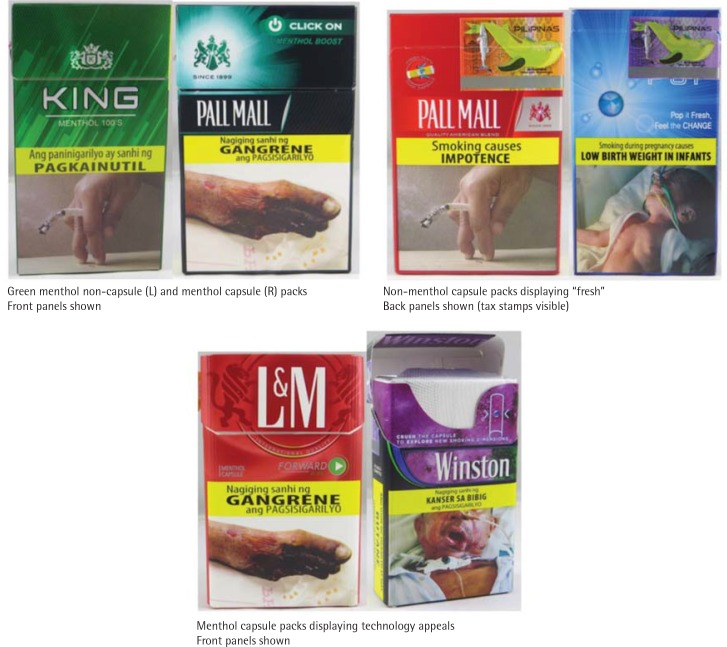
Examples of graphic components

Groups of packs varied significantly by a number of graphic components including color, the use of the descriptor ‘fresh’, and technology appeal (Table 3, Figure 2).

**Table 3:** Graphic components of cigarette packaging by packaging category, as distinguished by flavor and flavor capsule presence

*Descriptors*	*Group 1 Non-flavored non-capsule (n=37) n (%)*	*Group 2 Menthol non-capsule (n=23) n (%)*	*Group 3 Menthol capsule (n=10) n (%)*	*Group 4 Non-menthol capsule (n=6) n (%)*	*Groups 1 vs 2 p*	*Groups 1 vs 3 p*	*Groups 1 vs 4 p*	*Groups 2 vs 3 p*	*Groups 2 vs 4 p*	*Groups 3 vs 4 p*
Green as principal color and/or descriptor	1 (2.8)	21 (91.3)	6 (60)	2 (33.3)	<0.001*	<0.001*	0.049	0.053	0.008	0.608
Blue as principal color and/or descriptor	9 (25.0)	2 (8.7)	5 (50)	2 (33.3)	0.174	0.242	0.644	0.016	0.180	0.633
Purple as principal color and/or descriptor	0 (0)	0 (0)	2 (20)	0 (0)	-	0.043	-	0.085	-	0.500
Feminine appeal	0 (0)	0 (0)	0 (0)	0 (0)	-	-	-	-	-	-
Less harm descriptors	1 (2.8)	1 (4.3)	0 (0)	0 (0)	1.000	1.000	1.000	1.000	1.000	-
‘Taste’	3 (8.3)	1 (4.3)	2 (20)	2 (33.3)	1.000	0.295	0.141	0.212	0.100	0.604
‘Fresh, freshness, refresh’	0 (0)	0 (0)	1 (10)	3 (50)	-	0.217	0.002*	0.303	0.005*	0.118
‘Cool, ice, cold, chill, frost’	0 (0)	1 (4.3)	3 (30)	1 (16.7)	0.390	0.008	0.143	0.073	0.377	1.000
‘Pleasure, satisfaction, enjoyment, relaxing’	0 (0)	0 (0)	0 (0)	1 (16.7)	-	-	0.143	-	0.207	0.375
‘Sensation’	0 (0)	1 (4.3)	2 (20)	0 (0)	0.390	0.043	-	0.212	1.000	0.500
Technology descriptors or imagery	3 (8.3)	1 (4.3)	10 (100)	6 (100)	1.000	<0.001*	<0.001*	<0.001*	<0.001*	-

* p<0.008

a critical value of 0.008 was used to assess whether any pairwise comparison was considered statistically significant in order to account for multiple comparisons and control the family-wise error rate. A dash (-) indicates no difference between groups.

#### Color

A total of 30 packs displayed green as a principal color on the pack and/or displayed the descriptor ‘green’. A significantly greater proportion of menthol non-capsule packs (n=21; 91.3%) and menthol capsule packs (n=6; 60%) displayed green as a principal color or as a descriptor than non-flavored non-capsule packs (n=1; 2.8%) (p<0.001 for both comparisons). A total of 18 packs displayed blue as a principal color on the pack and/or displayed the descriptor ‘blue’; nine (25.0%) were non-flavored non-capsule packs, two (8.7%) menthol non-capsule packs, five (50%) menthol capsule packs, and two (33.3%) non-menthol capsule packs. There were no significant differences in the proportion of packs displaying blue as a principal color or as a descriptor across groups. Two packs displayed purple as a principal color on the pack and/or displayed the descriptor ‘purple’ or a specific shade of the color purple; both packs were menthol capsule packs (33.3%).

#### Less harm descriptors

Two packs displayed descriptors that indicate less harm (e.g. light, mild, low, safe, smooth, soft, mellow); one non-flavored non-capsule pack (2.8%) and one menthol non-capsule pack (4.3%). In one pack, the cigarette stick displayed the descriptor ‘mild’ and one pack displayed the term ‘smooth taste’.

#### Taste or sensation descriptors

Eight packs displayed the descriptor ‘taste’ on the pack; three (8.3%) non-flavored non-capsule packs, one (4.3%) menthol non-capsule pack, two (20%) menthol capsule packs, and two (33.3%) non-menthol capsule packs. Four packs displayed the descriptors ‘fresh’, ‘freshness’ and/or ‘refresh’. A significantly greater proportion of non-menthol capsule packs (n=3; 50%) displayed ‘fresh’, ‘freshness’ and/or ‘refresh’ compared to non-flavored non-capsule packs and menthol non-capsule packs (n=0 for both groups; p=0.002 and p=0.005, respectively). Five packs displayed the descriptors ‘cool’, ‘ice’, ‘cold’, ‘chill’ and/ or ‘frost’; one (4.3%) menthol non-capsule pack, three (30.0%) menthol capsule packs, and one (16.7%) non-menthol capsule pack. One (16.7%) non-menthol capsule pack displayed the descriptors ‘pleasure’, ‘satisfaction’, ‘enjoyment’ and/or ‘relaxing’. A total of three packs displayed the descriptor ‘sensation’; one (4.3%) menthol non-capsule pack and two (20.0%) menthol capsule packs.

#### Feminine appeal

No packs were identified as having a specific feminine appeal.

#### Technological appeal

Twenty packs (26.3%) displayed technology descriptors or imagery. A significantly greater proportion of menthol capsule packs (n=10; 100%) and non-menthol capsule packs (n=6; 100%) displayed technology descriptors or imagery than non-flavored non-capsule packs (n=3; 8.1%) and menthol non-capsule packs (n=1; 4.3%) (p<0.001 for all comparisons).

## DISCUSSION

Our findings demonstrate that different structural elements and graphic components are used on cigarette packaging to distinguish cigarettes with menthol and flavor capsules in the Philippines. The use of hard packs to store flavor capsule packs is not reported elsewhere, but is not surprising given that hard packs offer better protection to cigarettes than a soft pack, resulting in a lower chance that the capsules in cigarettes will be crushed before the user decides to do so. Additionally, hard packs connote quality by communicating prestige and expense; tobacco industry documents reveal that hard packs are preferred by females and that they are perceived by consumers to contain less harsh cigarettes than soft packs^25^. The use of green packaging to market menthol cigarettes support tobacco industry conclusions that users associate green coloring with menthol cigarettes^30^. The use of the descriptor ‘fresh’ or similar descriptors to suggest menthol or mint flavoring is in line with other observations of flavor capsule cigarettes^7^. While ‘fresh’ and similar descriptors have been associated with menthol flavoring, it is important to note that in our sample the descriptor was paired with the descriptor ‘menthol’ on only one pack and on the remaining three packs, printed on packs that did not name a characterizing flavor. It is likely that these three packs are menthol flavored, but this would have been confirmed through analysis of the cigarette ingredients. The use of descriptors such as ‘cold’, ‘ice’, ‘chill’ and ‘frost’ on menthol and flavor capsule packaging also supports what has been observed on menthol flavor capsule packaging^7^. ‘Ice’ was prominently used on Marlboro menthol capsule packs in our sample. The finding that no packs were identified as having a specific feminine appeal is notable given that the tobacco industry has been known to target females through marketing. This may be a reflection of the disparities in smoking prevalence among males and females in the Philippines where 41.9% of the male and only 5.8% of the female adult population smokes^5^. It may also be that our coding framework is not sufficiently sensitive or culturally tailored to detect feminine appeal of packs sold in the Philippines.

The use of technological appeals on flavor capsule packaging, regardless of flavor, was ubiquitous. Flavor capsule packs commonly displayed: button images similar to ‘play’ buttons or ‘fast forward’ buttons used on electronic devices; illustrations of the flavor capsule itself sometimes featured with an illustration of how it is situated inside the filter; and phrases such as ‘switch’ and ‘activate’. The use of these technological descriptors and imagery could be used to communicate innovation to consumers. Innovation is recognized by the tobacco industry as a key element to product growth and a strategy for maintaining positive consumer perceptions of brands^7^. Innovation in tobacco products serves three purposes: 1) justifies a higher price, 2) provides a different experience to the customer, and 3) suggests less risk^33^. It is plausible that the industry is using various technology descriptors and imagery to communicate the innovation of flavor capsules. Given that the youth and young adult populations are particularly aware of innovations in technology, we hypothesize that these appeals may partially explain some previous findings that conclude flavor capsule cigarettes are attractive to youth and associated with an interest to try^29^. We hypothesize that these findings may also extend to young adults.

While we did not distinguish between number of flavor capsules in each cigarette in our analysis, we observed two packs with cigarettes that contained two flavor capsules. These packs were categorized as menthol capsule packs in our analysis. Mevius Option Duo contained two menthol capsules described as ‘high menthol’ and ‘flavored menthol’. Marlboro Ruby Burst instructs the user to ‘crush the purple capsule for a burst of flavor’ and to ‘crush the green capsule for a boost in menthol taste’. In other countries, packs with up to four capsules in each cigarette have been observed^34^. It may be that the tobacco industry is using multiple capsules to give consumers more flavor options in response to their positive reaction to being able to customize the product, as well as pique curiosity among consumers with such unique offerings.

Structural elements and graphic components of cigarette packaging are used to create associations between the product and product characteristics in the mind of the consumer. Prior research has shown how the color green and descriptor ‘green’ have been used to convey menthol flavoring following a ban on menthol flavoring in Canada^35^ and how descriptors such as ‘smooth’ and ‘fine’ and colors such as white and gold have been used to connote a less harmful product following bans on misleading descriptors such as ‘light’, ‘mild’ and ‘low’^36,37^. Given our findings and what has been written previously on the marketing of menthol cigarettes, descriptors such as ‘fresh’ and ‘ice’, which are likely being used by the industry to communicate menthol flavoring, could potentially hinder tobacco control efforts by conveying flavor and by making it easy for the consumer to identify their original brand in the case that flavors or specific descriptors are banned.

These findings highlight the need for a greater awareness of the ability of cigarette packaging to convey product characteristics to consumers, some that may be misleading or that are attractive to a new generation of smokers. By highlighting the key packaging components that distinguish varieties of cigarettes, we can identify the specific marketing elements that influence consumer perceptions in future research. Indeed, our current research explores consumer perceptions of cigarettes that vary by structural elements and graphic components and assesses whether different cigarette packs distinguished by flavor and capsule inclusion are associated with attractiveness, less harm and intention to try menthol and flavor capsule cigarettes among young adults in the Philippines. Additionally, identifying packaging components that are used by the tobacco industry to denote a flavor or characteristic of a cigarette and can be used by consumers to identify their regular cigarette even after bans on flavors or specific descriptors (as has occurred in jurisdictions with bans on flavors and misleading descriptors^35,36^) can strengthen the call for plain packaging. The market share for capsule cigarettes increased between 2014 and 2017 in 57 of the 67 countries where they are sold, and in 2017 were the fastest growing segment in combustible tobacco^10^. The findings from this study will inform public health interventions in the Philippines and in 66 other countries in which flavor capsule cigarettes are sold, as well as in jurisdictions that are considering policies to address flavored tobacco.

### Strengths and limitations

There are several strengths and limitations in this study. To our knowledge, this is the first study to describe the differences in packaging between menthol and non-menthol cigarettes in any country. We also collected data in geographically and culturally diverse urban areas of the Philippines. However, because packs were exclusively gathered in highly populated cities in the Philippines, it is possible that our collection does not include some packs that are primarily sold in rural areas of the country, potentially biasing the sample. Limitations also include the age of our sample; it is likely that new packaging has been introduced to the market since late 2016. We can confirm that one change to the market is British American Tobacco’s withdrawal from the Philippines market at the end of 2017^7^. It is also possible that our coding scheme did not capture all components that might appeal to certain demographics, e.g. slim packs are found to appeal to females^25^ and it is possible that purple might appeal to females. However, this was not captured under our definition of feminine appeal. Additionally, we are not able to confirm the meaning of structural elements and graphic components to consumers in the Philippines, as this study did not include an assessment of consumer perceptions.

## CONCLUSIONS

This study identified structural elements and graphic components that are used on cigarette packaging to convey flavor and presence of flavor capsules. Further research should monitor the sale of flavor capsule cigarettes and use of flavors to attract new smokers, globally. Findings can inform future tobacco control policy as the Philippines and other countries consider bans on flavored tobacco, displays at point-of-sale, and adoption of plain tobacco packaging.
